# *C. elegans dss-1 *is functionally conserved and required for oogenesis and larval growth

**DOI:** 10.1186/1471-213X-8-51

**Published:** 2008-05-09

**Authors:** Johanna Pispa, Susanna Palmén, Carina I Holmberg, Jussi Jäntti

**Affiliations:** 1 Cellular Biotechnology Research Program, Institute of Biotechnology, University of Helsinki, Finland; 2 Molecular Cancer Biology Program, and Institute of Biomedicine, Biomedicum Helsinki, University of Helsinki, Finland

## Abstract

**Background:**

Dss1 (or Rpn15) is a recently identified subunit of the 26S proteasome regulatory particle. In addition to its function in the protein degradation machinery, it has been linked to BRCA2 (breast cancer susceptibility gene 2 product) and homologous DNA recombination, mRNA export, and exocytosis. While the fungal orthologues of Dss1 are not essential for viability, the significance of Dss1 in metazoans has remained unknown due to a lack of knockout animal models.

**Results:**

In the current study deletion of *dss-1 *was studied in *Caenorhabditis elegans *with a *dss-1 *loss-of-function mutant and *dss-1 *directed RNAi. The analysis revealed an essential role for *dss-1 *in oogenesis. In addition, *dss-1 *RNAi caused embryonic lethality and larval arrest, presumably due to loss of the *dss-1 *mRNA maternal contribution. DSS-1::GFP fusion protein localised primarily in the nucleus. No apparent effect on proteasome function was found in *dss-1 *RNAi treated worms. However, expression of the *C. elegans dss-1 *in yeast cells deleted for its orthologue *SEM1 *rescued their temperature-sensitive growth phenotype, and partially rescued the accumulation of polyubiquitinated proteins in these cells.

**Conclusion:**

The first knockout animal model for the gene encoding the proteasome subunit DSS-1/Rpn15/Sem1 is characterised in this study. In contrast to unicellular eukaryotes, the *C. elegans dss-1 *encodes an essential protein, which is required for embryogenesis, larval growth, and oogenesis, and which is functionally conserved with its yeast and human homologues.

## Background

Dss1 (or Rpn15) is a small (70-amino-acids in humans), conserved protein that has been implicated in several cellular functions. Although it was originally isolated as a candidate gene for the split-hand-split-foot syndrome (*DSS1*, deleted in the split hand/split foot SHFM1) [1], the connection with the syndrome has not been confirmed [2,3]. Instead, it has emerged that Dss1 is a subunit of the 26S proteasome [4-7]. The proteasome is a multi-protein complex responsible for the degradation of polyubiquitinated target proteins. It consists of a 20S core, responsible for the degradation of proteins inside the core channel, and a 19S regulatory particle, containing about 20 proteins arranged into a base and lid structure. The 19S regulatory particle binds ubiquitin-tagged substrates, unfolds the substrates and transfer them into the proteolytic core, but the precise role of many of the subunits is not yet known [8,9]. Within the cell, protein degradation must be regulated with precision. Emerging data indicates that the number of proteasomes and their subunit composition can vary depending on cellular conditions [9]. Various proteasome-interacting proteins have been discovered, and post-translational modifications of proteasome subunits may have an effect on proteolytic activity. Alternative regulatory particles to 19S exist that possibly function in ubiquitin-independent proteolysis, and the proteasome has also been implicated in tasks separate from protein degradation, e.g. transcription [10,11].

Dss1 binds to the 19S regulatory particle both in mammalian cells and in fission and budding yeast [4-7]. This binding is possibly mediated by lid components Rpn3 and Rpn7, with separate binding sites in Dss1 for both proteins [12,13]. Genetic and biochemical data from yeast suggest that Dss1 is involved in the proteolytic function of the proteasome. Accumulation of polyubiquitinated proteins occurs in *sem1 *mutants, the Dss1 homologue in *Saccharomyces cerevisiae*. Synergistic interactions are seen between double mutants of *sem1 *and a lid subunit, *rpn10 *[4-6]. In addition to a role in protein degradation, Dss1 is involved in DNA repair. It binds to BRCA2, a breast cancer susceptibility gene product and a component of the homologous recombination machinery [14,15]. Loss of either BRCA2 or Dss1, both in mammalian and fungal cells, results in defects in homologous recombination [16,17]. Conflicting evidence exists whether the effect of Dss1 is mediated by regulation of BRCA2 stability [17,18]. It is possible though that the Dss1 effect on homologous recombination is dependent on its role in the proteasome complex as proteasomes have been shown to bind to double-stranded breaks, sites of homologous recombination, and specifically BRCA2 binds the proteasome lid components Rpn3 and Rpn7, similar to Dss1 [5,13]. Moreover, proteasome activity is required for DNA repair. However, Dss1 does not regulate BRCA2 affinity for the proteasome, as the BRCA2-Rpn7 interaction is not dependent on Dss1 [13]. Dss1 has also been shown to be required for mRNA export in fission yeast [19]. Finally, both *SEM1 *overexpression and deletion in *S. cerevisiae *can suppress exocyst mutations, suggesting that Dss1 is directly or indirectly involved in protein secretion [20].

Sem1 in yeast is dispensable at 24°C, the normal growth temperature of yeast, despite the accumulation of polyubiquitinated proteins. However, *SEM1 *deleted cells are temperature-sensitive ceasing to grow at elevated temperatures [14]. In addition, loss of Sem1 triggers or enhances a cell differentiation process, pseudohyphal growth, in diploid *S. cerevisiae *cells [20]. In higher eukaryotes, there is currently no *in vivo *data about the significance of Dss1. Attempts to identify mutations in the *DSS1 *coding region of a selected set of breast cancer patients did not support a direct role for Dss1 in tumorigenesis [21]. Here we present the first characterization of *dss-1 *in a multicellular organism, the nematode *Caenorhabditis elegans*. We show that *dss-1 *is functionally conserved from fungi to metazoans, and required for oogenesis and intestinal function. In addition, *dss-1 *is important for embryogenesis and larval growth. Taken together, the results show, for the first time, that *dss-1 *is essential for the development of metazoan animals.

## Results

### *C. elegans dss-1 *is expressed throughout development and encodes a nuclear protein

**Figure 1:**
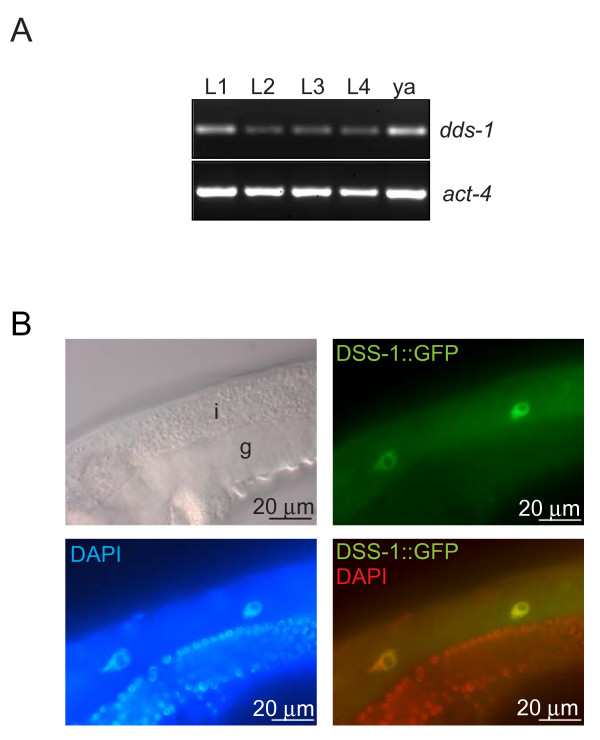
**Expression of *C. elegans dss-1***. A. *dss-1 *mRNA is expressed at different larval ages (L1 – L4) and in adult worms (ya, young adult) as shown by RT-PCR. *act-4*, actin (M03F4.2). B. DSS-1::GFP expressed under the *dss-1 *promoter localises to the nucleus of intestinal cells as shown by colocalisation with DAPI. g, gonad; i, intestine.

The *C. elegans dss-1 *(Y119D3B.15) encodes a 82 aa protein that is 55% similar and 34% identical to the product of the *S. cerevisiae SEM1 *gene, and 71% and 46%, respectively, to the human *DSS1 *gene product [20]. We examined the expression of the *dss-1 *transcript and DSS-1::GFP fusion protein in worm tissues. It has previously been shown that human and mouse *dss1 *are expressed ubiquitously in several adult and fetal tissues [1]. Similarly, the *C. elegans dss-1 *transcript was present throughout larval development and in adult worms (Figure 1A). To observe the cellular localisation of DSS-1, transgenic worms were generated expressing DSS-1::GFP fusion protein from 4 kb of *dss-1 *promoter sequence. This fusion protein localised to the intestinal epithelium and to unidentified neuronal cells in the head region (data not shown). No expression was seen in the gonad, presumably because of germ line silencing of transgene expression. Subcellularly the strongest expression was seen in the nucleus with diffuse staining in the cytoplasm (Figure 1B). This is similar to the reported Dss1 localisation in cultured human and yeast cells [14,22].

### *dss-1 *is required for fertility

**Figure 2:**
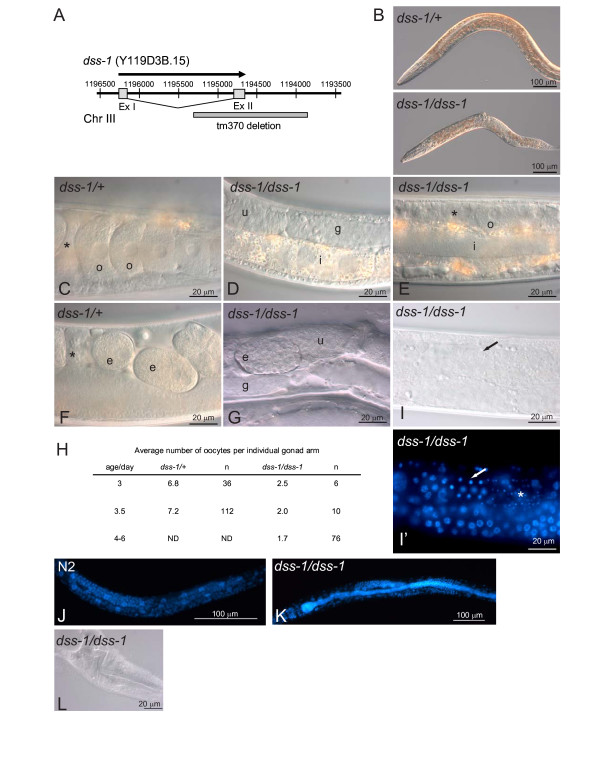
***dss-1 *deletion results in defects in oogenesis and intestinal absorption**. A. *dss-1 *gene is disrupted in the *tm370 *allele by a deletion spanning exon 2. B. *dss-1(tm370) *adults are sterile and thinner than control heterozygotes. Oocytes (C) and embryos (F) are visible in a control *dss-1 *heterozygote but *dss-1 *homozygotes have no (D) or only a few oocytes (E). G. Structures resembling embryos are occasionally seen in the uterus of *dss-1 *homozygotes. Asterisk, spermatheca; e, embryo or embryo-like structure; g, gonad; i, intestine; o, oocyte; u, uterus. H. The average number of oocytes in *dss-1 *homozygotes is reduced. Oocytes were identified either by Nomarski optics or by DAPI staining. n, number of individual gonad arms examined; ND, not determined. I. Spermatocytes (arrow) are still present in a *dss-1 *homozygote. I'. DAPI staining. Asterisk marks the more differentiated spermatids. J. Intestinal epithelial cell nuclei can be visualized by DAPI staining in a control N2 L4 worm. K. In *dss-1 *homozygotes an expanded intestinal lumen filled with bacteria can be seen. L. The pharynx is often twisted, possibly by pressure from the enlarged intestine.

To study the significance of *dss-1 *we examined the phenotype of *dss-1 *mutant worms. A deletion allele for *dss-1 *was obtained from the National Bioresource Project, Japan. *dss-1(tm370) *is a loss-of-function allele with a 1466 bp deletion completely removing exon 2 (Figure 2A). The *dss-1(tm370) *homozygous worms grew up into adults but produced no offspring (Figure 2B). We tested whether the lack of progeny were due to a defect in sperm formation by mating *dss-1 *mutant worms with N2 males. No offspring were obtained from mutant worms indicating that wild type sperm cannot rescue the sterility of *dss-1(tm370) *(n = 7). The sterility is not due to compromised gonad development as a two-arm gonad with developing germ cells was present in 98% of mutant worms examined (n = 169). Gonad development was further studied by crossing the mutants to the *lag2::GFP *marker strain. This marker expresses GFP in the distal tip cells (DTC) of the gonad [23]. Comparable numbers of *dss-1(tm370) *homo- and heterozygous worms showed a GFP signal in both distal tip cells (data not shown). However, the formation of oocytes was defective in the *dss-1(tm370) *homozygous worms (Figure 2C–E). Only 23% of 3-day-old mutant worms had any oocytes (n = 30) in contrast to 100% of the heterozygous worms of similar age containing oocytes (n = 18). The difference was partially due to a delay in oogenesis, as the percentage of mutant worms with oocytes increased to 50% when the worms were examined at 3.5 days (n = 72), and to 73% at 4–6 days after egg-laying (n = 67). However, the oocyte formation was abnormal as also in the older mutant worms the number of oocytes was always less than in control worms, with an average of two oocytes per gonad arm (Figure 2H). The oocytes were generally smaller and aberrant-looking. Occasionally embryo-like multicellular structures were visible in the uterus (25% in 3.5-day-old and 57% in 4–6-day-old worms) (Figure 2F and 2G). Their number per gonad arm was low, and no eggs were laid by the *dss-1(tm370) *mutants. Sperm were present in the spermatheca of the mutant worms in numbers comparable to the heterozygous control worms (83% of *dss-1(tm370)/dss-1(tm370) *mutants had sperm versus 94% *dss-1/+ *in 3-day-old worms). This suggested that spermatogenesis was superficially normal. However, some delay in sperm formation could be witnessed as spermatocytes were still found in 35% of 3-day-old *dss-1 *mutants, but in none of the control worms (Figure 2I and 2I'). In 3.5-day-old mutants spermatocytes were no longer seen.

The growth rate of the *dss-1 *mutants was examined by counting the progeny of individual *dss-1*/*sC1 *heterozygotes grown for 4 days (Table 1) (the *sC1 *balancer carrying the *dpy-1(s2170) *mutation was used to balance the *dss-1(tm370) *mutation. Homozygous *sC1 *worms were identified by the dumpy phenotype). No embryonic or larval lethality was evident. The expected ratios of fertile (*dss-1/sC1*, 48% vs. expected 50%) and dumpy adults (*sC1/sC1*, 26% vs. expected 25%) were observed. The remaining *dss-1(tm370)/dss-1(tm370) *worms were classified as either sterile or young adults indicating that the mutant worms seemed to grow at comparable rates to their heterozygous siblings. When grown at 25°C or 26°C, the results were similar except that the adult mutant worms were smaller than mutants grown at lower temperatures (data not shown). *dss-1(tm370) *homozygotes grown at room temperature were thinner than *dss-1/+ *heterozygotes, with often a slightly protruding vulva (Figure 2B). The lumen of their intestine was generally (82%, n = 138) expanded (Figure 2J and 2K) and filled with undigested bacteria giving the worms a somewhat shiny appearance. This was often accompanied by a twisted pharynx (Figure 2L). In *dss-1/+ *heterozygotes the intestinal lumen was also expanded in 36% of the worms examined (n = 78), but to a much milder degree than in the homozygotes. The lifespan of homozygous *dss-1 *mutants was shorter than that of heterozygous or wild type N2 controls (Table 2). At 25°C the earlier death of the mutants was slightly more pronounced.

**Table 1:** Growth rate of *dss-1/sC1 worms*

	***11 h***	***24 h***	***48 h***	***70 h***	***92 h***
embryo	87% (120)	3% (5)			
L1/L2	13% (18)	94% (149)	2% (4)		
L3		3% (4)	41% (67)	1% (2)	
dumpy L3			14% (24)	1% (1)	
L4			25% (41)	8% (14)	
dumpy L4			11% (19)		
young adult			4% (6)	14% (24)	9% (13)
dumpy			2% (3)		
young adult					
sterile adult				5% (8)	17% (26)
fertile adult				45% (76)	48% (71)
dumpy adult			1% (1)	26% (43)	26% (38)
n	138	158	165	168	148

The progeny of three individual *dss-1(tm370)/sC1 *worms were followed for four days. sC1 balancer carries the recessive dumpy-1 mutation. The results are a representative of three independent experiments.

**Table 2:** *dss-1 *mutants have a reduced lifespan

	***Day 1***	***Day 2***	***Day 3***	***Day 4***	***Day 5***
**RT**					
*dss-1/sC1*	100% (25)	100% (25)	100% (25)	100% (25)	88% (22)
*dss-1/dss-1*	100% (21)	86% (18)	86% (18)	10% (2)	0% (0)
					
**25**°**C**					
N2	100% (25)	100% (25)	100% (25)	92% (23)	84% (21)
*dss-1/sC1*	100% (25)	96% (24)	92% (23)	88% (22)	48% (12)
*dss-1/dss-1*	100% (25)	96% (24)	60% (15)	8% (2)	0% (0)

Young adult N2 and *dss-1 *homo- and heterozygous worms of equivalent age were placed at either RT or 25°C, and transferred onto fresh places when required. The number of survivors was followed for four days. The results are a representative of two (RT) and three (25°C) independent experiments.

### *dss-1 *is required for embryogenesis and larval growth

**Figure 3:**
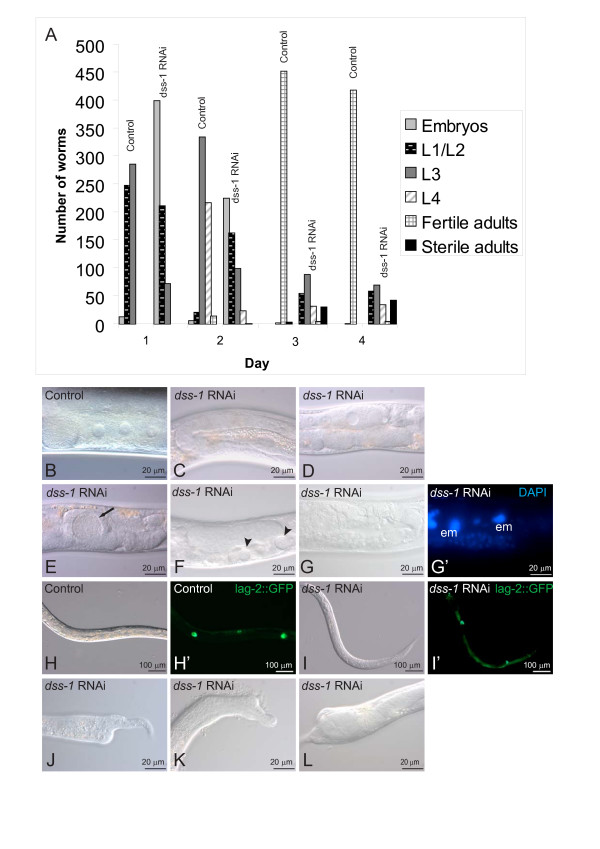
***dss-1 *RNAi results in sterility and growth defects**. A. *dss-1 *RNAi treated worms have higher embryonic lethality and larval arrest compared to vector-treated control worms. One representative of three separate 26°C RNAi experiments is plotted. F1 progeny were followed for four days after N2 L4 worms were placed on RNAi plates. B. Vector-treated control N2 worms have a normal number of oocytes, but in *dss-1 *RNAi treated worms the oocytes are absent (C) or present in reduced numbers (D). Embryo-like structures in the uterus (E, arrow), vacuoles (F, arrowheads), and *emo *cells (G, G', em) in the gonad are found occasionally. DTC cells are present in both vector- (H, H') and *dss-1 *RNAi (I. I') treated worms. Deformed tail (J) and head epithelia (K) are seen in worms arrested at early larval stages. L. Undigested bacteria are found in the bloated intestinal lumen.

RNAi against the *dss-1 *transcript was performed with bacterial feeding by placing N2 L4 larva onto plates. Phenotypes observed in the *dss-1 *RNAi F1 progeny mimicked the *dss-1(tm370) *phenotypes in a temperature-sensitive manner. When RNAi was performed at 15°C and 20°C no phenotype was evident. At temperatures between 24.5°C and 26°C sterile worms were found so that at lower temperatures only a fraction of the *dss-1 *RNAi treated worms were sterile (data not shown), whereas at 26°C no offspring were produced. At the same temperatures, but with vector-treated control RNAi, the worms were fertile (Figure 3A). Similar to the *dss-1(tm370) *mutants, oogenesis was defective with only 18% (n = 40) of the worms having oocytes (Figure 3B–D). Very few embryo-like structures were found in the uterus (Figure 3E). Instead, 32% of the gonads contained abnormal vacuolar structures (Figure 3F). DAPI staining revealed *emo*-like cells in the gonad (Figure 3G and 3G') and uterus. The *emo *phenotype is caused by endomitotic DNA replication resulting in polyploidy, and is identified by strongly DAPI positive nuclei. In order to follow gonadal development, RNAi was also performed in a *lag2::GFP *strain. Two DTC cells were found in equivalent numbers in both control (88%, n = 25) and *dss-1 *(88%, n = 65) RNAi worms (Figure 3H and 3I).

In addition to mimicking the *dss-1(tm370) *mutant phenotype, *dss-1 *RNAi treated worms had additional phenotypes, most likely due to depletion of the maternal transcript. At 26°C a major portion of the RNAi worms showed embryonic lethality and larval arrest (Figure 3A). Some of the worms arrested at L1/L2 stages and some later at L3/L4. 5–10% of the worms had deformed epithelia, either in the tail or the head (Figure 3J and 3K). These worms were usually arrested at L1 or L2. Those *dss-1 *RNAi worms that survived until L3 rarely had a deformed head or tail, and no *dss-1 *RNAi adult survivors had epithelial defects. Similar to the *dss-1(tm370) *mutants 40% of the surviving RNAi worms had a bloated intestine with undigested bacteria (Figure 3L).

### *dss-1(tm370) *phenotype partially mimics *brc-2 *and *kgb-1 *mutants

**Figure 4:**
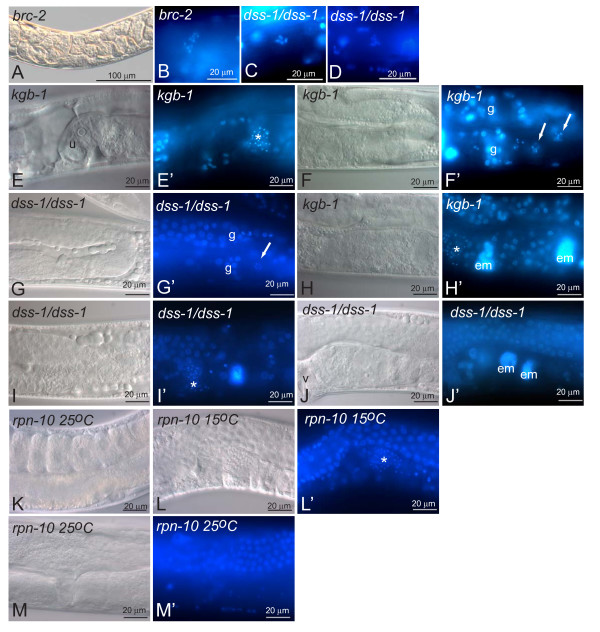
***dss-1 *phenotype resembles both *brc-2 *and *kgb-1 *mutants**. A. *brc-2(tm1086) *mutants are sterile with a uterus filled with abnormal embryos. B. Abnormal diakinesis occurs in *brc-2 *oocytes as shown by DAPI staining. C. *dss-1 *oocytes appear to have the expected number of six condensed chromosomes, but they occasionally show defects in diakinesis (D). In B-D only one focal plane is shown. E-M. Nomarski optics; E'-M' DAPI staining. E. *kgb-1 *mutants grown at 26°C lack embryos in the uterus (u). Their gonads are often disorganised (F, F', arrows indicate oocyte chromosomes) and similar disorganisation is sometimes seen in *dss-1 *homozygotes (G, G', arrow indicates an oocyte). DAPI-positive *emo *oocytes are often present in *kgb-1 *(H), and occasionally in *dss-1 *gonads (I). J. *emo*-like structures are also seen in *dss-1 *uteri. K-M. *rpn-10 *mutants grown at the restrictive temperature (25°C) have oocytes (K) but lack sperm (L, M). 15°C, permissive temperature. Asterisk, spermatheca; em, *emo *cell; g, germ cells; u, uterus; v, vulva.

As Dss1 has been shown to bind BRCA2 [14,15] we wanted to compare the mutant phenotype of the worm *BRCA2 *homologue *brc-2 *with *dss-1*. Similar to the *dss-1(tm370) *mutants the *brc-2(tm1086) *worms are sterile. However, unlike the *dss-1 *mutants they produce eggs, which, nevertheless, do not develop and are not laid (Figure 4A and [24]). During diakinesis *brc-2 *mutant oocyte chromosomes do not condense normally but aggregate instead (Figure 4B and [24]). In *dss-1 *mutants several oocytes have apparently six normally condensing chromosomes (Figure 4C), although we found a few with abnormal diakinesis (Figure 4D).

We examined Wormbase [25] for mutants with similar defects in oogenesis as in *dss-1 *mutants, and looked for proteins that might play a role in similar processes as DSS-1. We chose to study the *kgb-1 *mutant for comparison with *dss-1 *mutants for two reasons. First, the *kgb-1 *mutant phenotype has many similarities with the *dss-1 *homozygotes. The mutants are sterile, with no mature oocytes or embryos, and, in older worms, exhibit a protruding vulva and bloated appearance [26]. This sterility is temperature-sensitive at 26°C similar to the *dss-1 *RNAi phenotype. Secondly, KGB-1, a MAP kinase, is believed to act in proteolytic degradation of an oogenesis-specific protein. It binds GLH-1, an RNA helicase required for fertility [26], and in *kgb-1 *mutants GLH-1 protein levels are elevated [27]. Biochemical evidence indicates that KGB-1 regulates GLH-1 degradation by phosphorylation [27]. We postulated that DSS-1 and KGB-1 might both be regulatory factors required for the proteolysis of GLH-1 and/or other proteins required for oogenesis. *kgb-1(um3) *uteri were generally void of embryos (compare Figure 4E to Figure 2D). The gonad arms were disorganized with no well-formed rachis (data not shown). Occasionally the *kgb-1 *proximal gonad contained a mixture of more differentiated oocytes surrounded by less developed germ cells (Figure 4F). Although the *dss-1 *mutant gonads were in general more organized, upon closer inspection we found some similar mixtures of more mature oocytes surrounded by less mature germ cells (Figure 4G). A prominent feature of the *kgb-1 *mutant gonads is the *emo *phenotype (Figure 4H). In younger *dss-1 *mutants no DAPI-positive *emo *cells were visible either in the gonad or the uterus (n = 33). However, in older *dss-1 *mutants 20% of the gonads had *emo*-like cells (n = 24) (Figure 4I). In addition, in 71% of the *dss-1 *mutants DAPI-positive *emo*-like structures were found in the uterus, possibly resulting from unfertilised embryos (Figure 4J). In *kgb-1 *mutants 95% had *emo *cells in the gonad (n = 20), but only 20% in the uterus.

Examination of GLH-1 protein levels by Western analysis showed no significant differences in the amount of GLH-1 between *dss-1 *homozygotes, *dss-1/sC1 *heterozygotes, and N2 control worms (data not shown). This indicates that DSS-1 is not directly involved in the regulation of GLH-1 turnover.

### Proteasomal activity is unaffected by *dss-1 *RNAi

Most of the proteasome subunits are necessary for development, and their depletion by RNAi in *C. elegans *results in early lethality [28]. One exception is RPN-10, a component of the proteasome RP lid structure, which is not required for viability in yeast or *C. elegans *[29,30]. Instead, *rpn-10 *null worms are sterile at 25°C implying similarity to *dss-1(tm370) *mutants [30]. However, unlike *dss-1 *mutants, the main cause of sterility for *rpn-10 *mutants is the absence of sperm formation, while oocytes seem to form normally (Figure 4K–M and [30]).

In yeast *RPN10 *shows synthetic interactions with the *sem1/dss1 *deletion indicating involvement of both proteins in the same cellular process, protein degradation [4-6]. We tested whether similar genetic interactions between *dss-1 *and *rpn-10 *occur in *C. elegans*. With N2 or *rrf-3 *worms no effect was seen when RNAi against *dss-1 *was performed at 20°C. Similarly no phenotype was obtained either when *dss-1 *RNAi was performed at 20°C in an *rpn-10(tm1349) *background (data not shown), indicating a lack of a synthetic interaction. The experiment could not be done at higher temperatures due to the temperature-sensitive sterility of *rpn-10 *worms.

To examine whether DSS1 affects the proteolytic activity of the proteasome, the chymotrypsin-like activity of the proteasome was measured in whole worm extracts of *dss-1 *RNAi worms. Similar levels of activity were detected in extracts from *dss-1 *RNAi or control vector-treated worms. When the chymotrypsin-like activity was assigned as 100% (standard deviation 3.77 and 2.68 in two separate experiments) in extracts from control vector-treated worms, the activity in the *dss-1 *RNAi treated samples was 111.0% +/- 3.76 and 108.4% +/- 13.65, respectively. Accordingly, Western blot analysis revealed no difference in the levels of proteasome subunits in the *dss-1 *RNAi and control vector-treated worms (data not shown).

### *C. elegans dss-1 *can rescue *SEM1 *deletion defects in yeast

**Figure 5:**
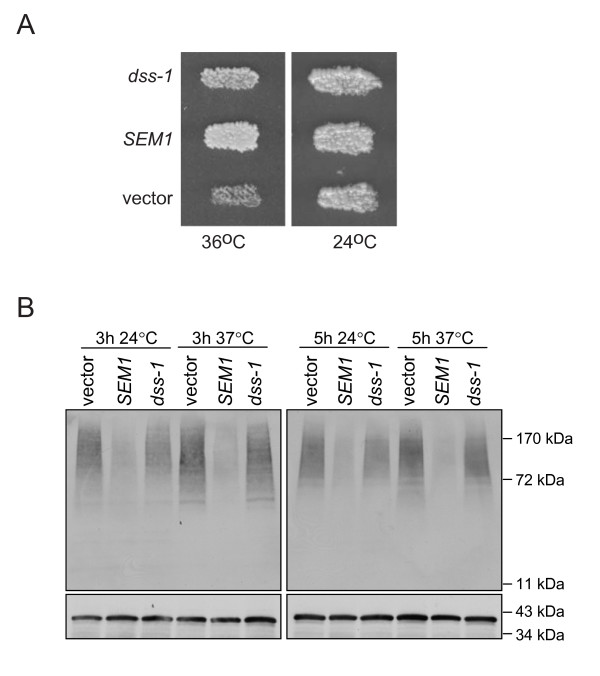
***C. elegans dss-1 *can partially rescue yeast *sem1 *mutation**. A. *S. cerevisiae sem1 *mutants have a growth defect at restrictive temperature (vector). Expression of either *C. elegans dss-1 *or the yeast *SEM1 *in *sem1 *mutants rescues the growth defect. B. Accumulation of polyubiquitinated proteins is seen in *sem1 *mutants transformed with an empty vector both at permissive and restrictive temperature at two different time points. Expression of yeast *SEM1 *in *sem1 *mutant cells reduces efficiently the accumulation of polyubiquitinated proteins, whereas expression of *C. elegans dss-1 *has only a modest effect. Lower panels have been blotted with anti-Sso1/2 antibody as a control for equal loading.

In order to further study the function of DSS-1, we made use of *S. cerevisiae *cells deleted for the *dss-1 *orthologue *SEM1*. These cells are temperature-sensitive for growth [14], and accumulate polyubiquitinated proteins [4,6]. Yeast cells deleted for *SEM1 *were transformed with an empty control plasmid or plasmids containing either *dss-1 *or the yeast *SEM1*. Transformants were tested for growth at elevated temperatures and for their ability to process polyubiquitinated proteins. The temperature-sensitive phenotype caused by the deletion of *SEM1 *was rescued both by the *C. elegans dss-1 *and by *SEM1 *(Figure 5A). At the same time a partial, but consistent rescue by the *C. elegans dss*-*1 *of accumulation of polyubiquitinated proteins was observed in *SEM1 *deleted cells (Figure 5B). Quantification showed that on average reintroduction of *SEM1 *to *sem1Δ *cells rescued 75–80% of the accumulated proteins whereas rescue by *dss-1 *was 50% after 3 h of incubation and 20% after 5 h (data not shown). Taken together, the growth phenotype and rescue from polyubiquitinated protein accumulation suggest that DSS-1 protein is functionally conserved.

## Discussion

Our analysis of the loss-of-function phenotype of *C. elegans dss-1 *shows that *dss-1 *is essential for the development of a multicellular animal. In *dss-1(tm370) *mutant animals oogenesis is severely affected, and food absorption of the intestinal membrane appears malfunctional. Depletion of the *dss-1 *transcript by RNAi results in increased embryonic lethality and larval growth arrest, indicating the importance of *dss-1 *also at early stages of nematode development.

### *dss-1 *and fertility

The germ line development in *dss-1(tm370) *animals was slightly delayed. At three days after egg-laying, control *dss-1 *heterozygotes had both mature oocytes and sperm, whereas *dss-1 *homozygotes still had spermatocytes and relatively few animals had oocytes at all. Approximately 12 h later sperm had matured in the majority of the mutants, and the proportion of adult worms with oocytes continued to increase. This delay could be intrinsic to the germ line itself. Alternatively, the sterility could be a secondary consequence caused by the general sickness of the worms, possibly mediated by the intestine. For example, lipoproteins secreted by the intestine are known to affect germ line development [31,32]. The 4 kb *dss-1 *promoter drives expression mainly in the intestine (Figure 1B). We attempted to rescue the *dss-1 *mutant with the DSS-1::GFP fusion protein expressed under the 4 kb promoter but were not successful (data not shown). Since transgenic expression is often silenced in the germ line [33] this suggests though that DSS-1 expression solely in the intestine is not sufficient for rescuing *dss-1 *oogenesis defects, and that its expression in the germ line may be required for oogenesis.

Although oocyte formation seemed to increase with aging of the *dss-1 *mutant worms it remained defective. Normal numbers of oocytes were not achieved, and the formed oocytes were not capable of fertilization, even with wild type sperm. *C. elegans *BRC-2 is required for proper progression of meiosis, and *brc-2 *mutants have abnormal diakinesis (chromosome condensation) [24]. Similarly, abnormal chromosome condensation was found occasionally in *dss-1 *mutants. However, abnormal diakinesis does not explain completely the oogenesis defects seen in *dss-1 *mutant worms as in *brc-2 *mutants oocytes progress through fertilization producing embryos that die, something not occurring to the same degree in *dss-1 *mutants. Thus, DSS-1 is likely to have a role in oogenesis independent from its function with BRC-2.

*kgb-1 *and *dss-1 *mutants have strongly overlapping phenotypes [26] and Figure 4), and both proteins have been linked to the protein degradation machinery. In *kgb-1 *mutants the increased levels of the GLH-1 RNA helicase appear to result in a defective germ line [27]. In *dss-1 *mutants GLH-1 levels were normal suggesting that *kgb-1 *and *dss-1 *are not involved in the same degradation process. It is possible that the oogenesis defects in *dss-1 *mutants might be caused by the aberrant regulation of degradation of another essential protein required for oogenesis, in combination with errors in homologous recombination.

### *dss-1 *is essential for growth and survival

RNAi against *dss-1 *causes growth defects and embryonic lethality not seen in *dss-1 *mutants. This could be caused by targeting of secondary mRNAs, homologous to *dss-1*. We find this explanation unlikely as no homologous targets have been identified either by Wormbase [25] or by our own attempts. An alternative possibility is that the progeny obtained from *dss-1 *RNAi treated worms are escapers from the oogenesis defect, and that the embryonic lethality and larval arrest observed are caused indirectly by partially defective oogenesis. However, as the parental generation of RNAi treated worms showed no signs of sterility and laid eggs comparably to the control worms (Figure 3A), we do think this is a probable option. Therefore, we believe that the depletion of the maternal transcript provided by the heterozygous mothers of *dss-1 *mutants results in the early defects seen in the RNAi treated worms. This suggests that the *dss-1 *transcript is an essential component of embryogenesis and larval development. It is feasible that these effects are mediated by the involvement of DSS-1 in proteasome function. The proteasome is required for development, as RNAi against the majority of the *C. elegans *proteasome subunits has a strong, early lethal effect [28].

Accumulation of bacteria in the intestine is seen in the *dss-1 *mutants and *dss-1 *RNAi treated worms. This could be caused by defective absorption by the intestinal membrane or alternatively, by problems in secretion of digestive enzymes. The fact that the yeast homologue of DSS-1, Sem1, has also been linked functionally to exocytosis suggests that DSS-1 participates directly or indirectly in secretion [20].

The *dss-1 *mutant worms have a slightly reduced lifespan. In addition, stress such as heat appears to enhance the phenotypic effects seen in *dss-1 *mutants or RNAi worms. This is in line with the role of DSS-1 in protein degradation since the proteasome function has previously been linked to aging and stress [9,34].

### The cellular function of DSS-1

Dss1 is known to participate in the proteasome complex, and either directly or indirectly via proteolytic activity in BRCA2 function and DNA repair [4-7,14,15]. In addition, it has been implicated in mRNA export and regulation of exocytosis [19,20]. What does the analysis of the loss-of-function phenotype of *dss-1 *in *C. elegans *tell us of its function in a multicellular organism?

The proteasome is an essential component of the housekeeping machinery inside a cell, and perturbing its function generally results in lethality [28,35]. As such, the significant portion of embryonic lethality and larval growth defects seen in *dss-1 *RNAi treated worms are indicative of an essential function. Whether this function relates to the proteasome is uncertain. We did not observe an apparent effect on proteasome activity or on the amount of proteasome subunits in whole worm extracts when *dss-1 *was knocked down by RNAi. In addition, *dss-1 *homozygous mutants survive into adulthood with only specific defects. The homozygous *dss-1 *mutant embryos are likely to be rescued by expression of the maternal *dss-1 *transcript. The lack of a lethal phenotype in the adult homozygotes can indicate either that DSS-1 is not required for proteasome activity in adults, or that the requirement of DSS-1 for proteasome activity is restricted to specific tissues or specific subsets of proteins. Indeed, recent work in cultured mammalian cells showed that depletion of *dss-1 *mRNA by RNAi has a minimal effect on proteolytic activity [36]. Taken into account that the *dss-1 *depletion in their experiment was not complete, it is possible that in metazoans Dss1 is not necessary for general proteasome activity. In order to resolve these issues, methods to measure local or substrate-specific proteasome activity in the *dss-1 *mutant worms are required.

The fertility defects seen in the *dss-1 *mutants differed from the defects seen when *rpn-10*, another proteasome subunit, was mutated [30]. Either, DSS-1 and RPN-10 have independent tasks in the proteasome complex, or, alternatively, RPN-10 acts redundantly with another subunit, thus explaining the mild oogenesis defects seen in *rpn-10 *mutants. Indeed, RNAi against *rpn-10 *has no effect, whereas combined RNAi against *rpn-10 *and *rpn-12 *is lethal [28]. We did not observe any synthetic effect between *rpn-10 *and *dss-1 *suggesting that RPN-10 and DSS-1 are not required for the same molecular process.

The function of *dss-1 *has been at least partially conserved during evolution, as we have shown that both the human and nematode homologues can complement the growth and differentiation phenotypes of the yeast *sem1 *mutation (Figure 5, [20]). However, the *C. elegans dss-1 *can not fully rescue the accumulation of polyubiquitinated proteins in *sem1 *mutant yeast cells. *sem1 *mutant cells accumulate polyubiquitinated proteins already at the permissive temperature but their growth compared to *SEM1 *expressing cells is indistinguishable. It is possible that the homology between DSS-1 and the yeast Sem1 protein is only sufficient for partial functionality in proteasome activity, but that even this incomplete activity is enough to sufficiently reduce the amount of polyubiquitinated proteins to allow growth at the restrictive temperature. Alternatively, the temperature-sensitive growth defect could result from a proteasome-independent function of Sem1 that is not conserved between yeast and nematode. We do not think that this function would be related to homologous recombination. First, in yeast no BRCA2 homologue has been identified. Second, loss of *SEM1 *in *S. cerevisiae *does not cause increased sensitivity to UV-irradiation or to chemicals that induce double-stranded breaks, or cause significant differences in the capability of these cells to repair gapped plasmids ([5], J. Jäntti, unpublished). It is thus likely that the growth complementation in yeast is based on functions of DSS-1 in processes other than homologous recombination.

## Conclusion

*C. elegans dss-1 *is functionally conserved with its yeast and human homologues. Although no direct effect on the proteasome function was observed in the *dss-1 *knockdown worms, the efficient growth rescue and the partial rescue of accumulated polyubiquitinated proteins in yeast suggest that *C. elegans *DSS-1 is likely to play a role in proteasome function. It is possible that this role is spatially or temporally restricted. In summary, in contrast to unicellular organisms, *dss-1 *in a multicellular animal is an essential gene, which is required for oogenesis, embryogenesis, and larval growth.

## Methods

### Genetics

Worms were grown at room temperature on NGM plates unless otherwise indicated [37].

The following worm strains used:

Bristol N2 (CGC)

*rrf-3(pk1426) *(gift from Simon Tuck)

*dss-1(tm370)/+ *(Shohei Mitani, NBP, Japan)

*sC1(s2023) [dpy-1(s2170)] *(Genetic Toolkit project, BC4279, CGC)

*brc-2(tm1086)*/*hT2 *(DW104, CGC)

*kgb-1(um3) *(KB3, CGC)

*rpn-10(tm1349) *(Shohei Mitani, NBP, Japan)

*lag-2::GFP *(qIs56 IV or V, JK 2868, CGC)

*pie-1::GFP::H2B *(ruIs32 III, AZ212, CGC)

*vit-2::GFP *(*sqt-1(sc103), bIs7) *[31] (gift from Simon Tuck)

*dss-1(tm370) *allele was outcrossed once when balanced against *sC1*. Three types of worms were obtained from the balanced strain: sterile *dss-1 *homozygotes, fertile *dss-1*/*sC1 *heterozygotes, and fertile dumpy *sC1 *homozygotes. For crosses of *dss-1(tm370) *mutants with wild type males, L3 or L4 non-dumpy larvae from the progeny of *tm370/sCi *heterozygote worms were mated on individual plates with three N2 males each. The 52 larvae tested were either hetero- or homozygous for *dss-1*. After scoring for the presence of progeny, the parent hermaphrodite was genotyped by PCR.

### Plasmids

*dss-1::dss-1::GFP *transgenic construct was made from YAC Y119D3 by PCR of 4 kb of 5' *dss-1 *promoter sequence together with *dss-1 *ORF. Two adjacent PCR fragments: 1) a 2.6 kb *dss-1 *promoter fragment (oligos 5'-GCATTGCATGCGCTCACCGAGCATTGGAACAGAGG-3' and 5'-CCTCCATTCTCCGTCGACTACGGC-3') and 2) a 2.9 kb *dss-1 *promoter and ORF fragment (oligos 5'-GCCGTAGTCGACGGAGAATGGAGG-3' and 5'-GCATTGGATCCTCGGCGACTTGGTGTCCAGATTTGCG-3'), were cloned into *Sph *I/*Sal *I and *Sal *I/*Bam *HI sites of pPD95.67 containing the *GFP *gene (NLS site deleted with *Kpn *I) (Andrew Fire), respectively. Transgenic worms were generated by co-injections with plasmid pRF4 into the gonads of young adult N2 hermaphrodites, with F1 transgenics selected by their roller phenotype.

### RNAi

For *dss-1 *RNAi construct, *dss-1 *cDNA was cloned from yk115f10 (Yuri Kohara) into *Pst *I/*Xho *I sites of L4440 (Andrew Fire). RNAi was performed by bacterial feeding [38] either in Bristol N2, *rrf-3(pk1426)*, or *rpn-10(tm1349) *strains. Both L4 and L1 larvae were used as starting stages for the RNAi experiments. RNAi starting with L1 larvae were tested at different temperatures, but no phenotypes were observed. Phenotypes were obtained using N2 L4 larvae at 24.5°C – 26°C. At 26°C the RNAi phenotype was most severe (without the control worms being affected) so this temperature was chosen for phenotypic analysis. L4 larvae were placed on bacterial RNAi plates and allowed to lay eggs for 24 h after which they were transferred to a fresh set of RNAi plates. After an additional 24 h the worms were removed from RNAi plates. The progeny from both plate sets was followed for three to five days.

### PCR genotyping of *dss-1(tm370)*

Nested PCR was performed from single lysed worms. For identification of the *tm370 *allele, oligos designed by the National Bioresource Project, Japan [39] were used. For detection of the wild type allele the following oligos were used: external, 5'-ATCTACCGCCGCCAAAAAAG-3' and 5'-GATATCTTAGGCGACTTGGTGTCC-3'; internal, 5'-TCGGGAAAATAGGTTTTTAGGC-3' and 5'-TTCCTCCTTCAATTGCTTCG-3'. The wild type allele gave a circa 730 bp product, while no product was obtained from the *tm370 *allele.

### RT-PCR

For RT-PCR of staged N2 worms, the worms were synchronized at the L1 larval stage by alkaline hypochlorite treatment, spotted onto OP50 seeded NGM agar plates, grown at 20°C for 6 h (L1), 18 h (L2), 30 h (L3), 42 h (L4), or 66 h (young adult), and then harvested after visual inspection. Total RNA was extracted using TRIzol reagent (Invitrogen) and DNase treated using DNA-free kit (Ambion). Equal amounts of RNA (1.6 μg) were used to prepare cDNA with the First-Strand cDNA Synthesis Kit (Amersham BioScience) and the *Not *I-d(T)_18 _bifunctional primer according to the manufacturer's instructions. cDNA was amplified by standard PCR for 30 cycles using the forward primers 5'-TGCTGTCGTTGAGAAGAAGG (*dss-1*) and 5'-AAGGTGTGATGGTCGGTATGGG-3' (*act-4*) and the reverse primers 5'-GCGACTTGGTGTCCAGATTT-3' (*dss-1*) and 5'-TTCGTAGATTGGGACGGTGTGG-3' (*act-4*). Equal amounts of each reaction were then analyzed on 1% TBE agarose gel.

### DAPI staining

As the *dss-1 *mutant gonads were very fragile, either disintegrating or remaining inside the worm carcass upon dissection, DAPI staining was performed on whole animals. Worms were fixed in 3% PFA and postfixed in methanol. DAPI (Molecular Probes, USA) was used at 0.5 μg/ml.

### Proteasome activity assay

A fluorogenic peptide substrate assay was performed to determine the chymotrypsin-like peptidase activity of the proteasome in whole worm extracts. Whole worm extracts were prepared from frozen worm pellets resuspended in lysis buffer (50 mM HEPES pH 7.4, 150 mM NaCl, 5 mM EDTA, and 2 mM DTT) and subjected to sonication and centrifugation. 25 μg of whole animal extracts was incubated with 140 μM Suc-LLVY-AMC (Calbiochem) in proteasome activity assay buffer (50 mM HEPES pH 7.4, 150 mM NaCl, 5 mM EDTA, and 5 mM ATP) at room temperature.

The fluorescence intensity was measured in triplicate samples at 380 nm excitatory and 455 nm emission wavelengths using a FLUOstar Optima Mircoplate Reader (BMG Labtech). The assay was performed in the absence and presence of the proteasome inhibitor MG132 (10 μM, Peptides Interrnational) to calculate the proteasome-specific activity, after which the L4440 sample was assigned an activity value of 100%. The results are from two independent RNAi experiments.

### Western blotting

For GLH-1 immunoblotting 50 adult worms of each genotype were lysed in 25 μl of 1× Laemmli sample buffer. The samples were run on a 12% SDS-PAGE gel and transferred onto a nitrocellulose membrane. Rabbit anti-GLH-1 antibody (kind gift from Susan Strome) was used at a 1:1000 dilution. The membrane was reblotted with mouse anti-α-tubulin (Sigma T5168, dilution 1:1000) to check for equal loading. For detection of proteasome subunits in RNAi-treated worms, extracts were prepared from frozen worm pellets by sonication in lysis buffer supplemented with 0.5 mM NEM, 10 μM MG132 and complete protease inhibitor cocktail (Roche Diagnostics). Equal amounts of proteins were subjected to Western blotting with antibodies against proteasome α-subunits (BIOMOL International, PW8195) and α-tubulin.

### Yeast *sem1 *mutant rescue

Yeast strain H2187 (*MAT***a ***ura3 sem1::kanMX*) was transformed with the empty yeast expression plasmid pVT102U or pVT102U containing *dss-1 *or *sem1 *cDNA. Transformants were patched on SCD-ura plates, grown overnight at 24°C and replicated using velvet to different temperatures. The growth of the patches was followed for three days. For analysis of accumulation of polyubiquitinated proteins, the above transformants were grown overnight in SCD-ura at 24°C. The cultures were diluted to OD_600 _0.2 and regrown to OD_600 _0.6–1.0. The cultures were divided in two and the growth was continued either at 24°C or at 37°C. Samples were collected at 1.5, 3 and 5 hours after the temperature shift. Cells were lysed in 2% SDS, the total protein concentration was measured (BCA Protein Assay Kit, Pierce) and equal amounts of the proteins were subjected to SDS-PAGE and Western blotting with an anti-polyubiquitin antibody (BIOMOL International, PW8805) and anti-Sso1/2 antibody [40]. Quantification of the band intensities was done by scanning the films with GS-710 densitometer (Bio-Rad) using the Quantity One program, version 4.0 (Bio-Rad). The optical density of the whole lane for each sample was quantified. The polyubiquitin band intensities were normalized to the Sso1/2 band intensities.

## Authors' contributions

JP performed the mutant, RNAi, and anti-GLH-1 Western analysis; SP performed the *lag2::*GFP analysis, CIH designed and performed the developmental RT-PCR, anti-polyubiquitin Western, and proteasome activity assays; while JJ did the transgenic injections, yeast rescue assay, and conceived the study. Plasmid cloning was done by JP, SP, and JJ. The manuscript was written by JP, with revisions by CIH and JJ. All the authors read and approved the manuscript.
